# Supplementary material for: Multi-objective Bayesian active learning for MeV-ultrafast electron diffraction
Source: Nat Commun. 2024 Jun 3;15:4726. doi: 10.1038/s41467-024-48923-9 (PMC11148007; doi:10.1038/s41467-024-48923-9)
Supplement: Supplementary file 1 — Supplementary Information [file 41467_2024_48923_MOESM1_ESM.pdf]

# Supplementary Information

# Multi-Objective Bayesian Active Learning for MeV-ultrafast electron diffraction

Fuhao Ji∗, Auralee Edelen, Ryan Roussel, Xiaozhe Shen, Sara Miskovich, Stephen Weathersby, Duan Luo, Mianzhen Mo, Patrick Kramer, Christopher Mayes, Mohamed A. K. Othman, Emilio Nanni, Xijie Wang, Alexander Reid, Michael Minitti, Robert Joel England†

SLAC National Accelerator Laboratory, Menlo Park, 94025, California, USA.

Corresponding author(s). E-mail(s): *fuhaoji@slac.stanford.edu;

† england@slac.stanford.edu;

| Initial Pulse Charge (fC) | Range of $B_1$(T) | Range of $B_2$ (T) | $\phi$ (deg) |
|---|---|---|---|
| 10 | [0.13, 0.16] | [0.255, 0.364] | 55 |
| 50 | [0.13, 0.16] | [0.255, 0.326] | 55 |
| 100 | [0.13, 0.16] | [0.255, 0.326] | 55 |

Supplementary Table 1. Parameter range used in spot size vs q-resolution optimizations.

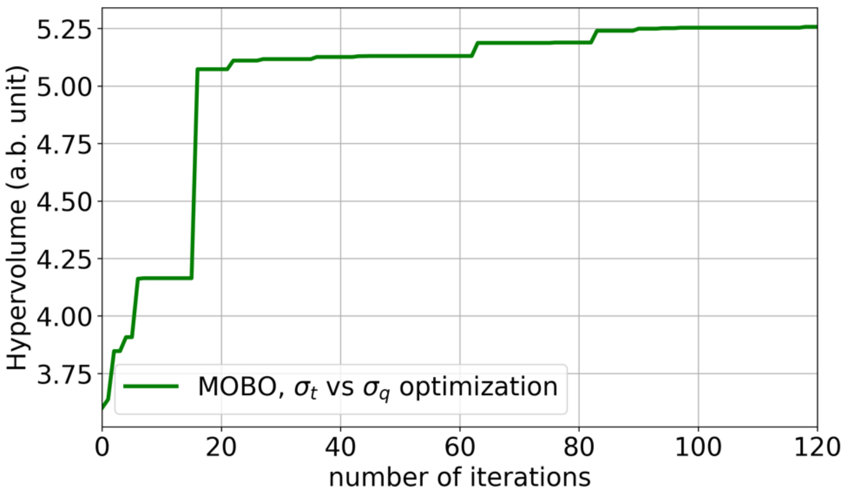


Supplementary Figure 1. Hypervolume convergence plot for the temporal length vs q-resolution optimization results.

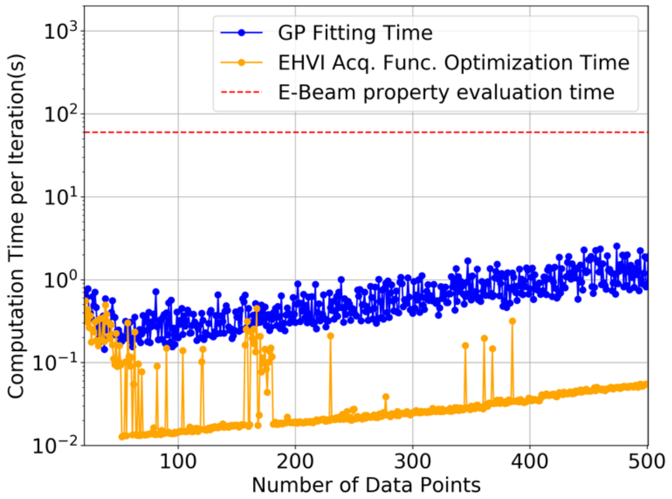


Supplementary Figure 2. Computation time per iteration during the MOBO process under the temporal length vs q-resolution optimization condition. For each iteration, the GP fitting time (blue) and EHVI acquisition function optimization time (orange) are below 5 s with < = 500 data points. The average time taken to measure electron beam properties is 60 s (shown as red dashed line in the figure).
